# Trends in incidence, mortality, and lethality due to meningitis in children and teenagers in Brazil: a nationwide time-series study from 2002 to 2022

**DOI:** 10.1590/1980-549720250047

**Published:** 2025-09-19

**Authors:** Alessandro Vidal de Oliveira, Geovanna Barros Rocha, Ana Luiza Nepomuceno Sampaio, Ana Lucy Peixoto Rabelo

**Affiliations:** 1 Universidade do Estado do Pará, Faculdade de Medicina - Belém (PA), Brazil.

**Keywords:** Meningitis, Health information systems, Incidence, Mortality, Epidemiology, Meningite, Sistemas de informação em saúde, Incidência, Mortalidade, Epidemiologia

## Abstract

**Objective:**

The aim of this study was to analyze the trends in incidence, mortality, and lethality rates due to meningitis in children and teenagers aged 0-19 years in Brazil and its regions by sex from 2002 to 2022.

**Methods:**

This is a time-series study of the cases and deaths due to meningitis with data from the Notifiable Diseases Information System. We estimated the average annual percent change (AAPC) and its 95% confidence interval (95%CI) via Joinpoint regression, in addition to comparing the Brazilian Federative Units’ AAPCs by a pairwise coincidence test.

**Results:**

There were 274,500 cases and 17,162 deaths during the analyzed period, with downward trends both in Brazil and its regions regarding the incidence and mortality rates. In Brazil, the lowest AAPC in incidence was among males (AAPC=-5.7, 95%CI -7.1 to -4.2), whereas the lowest AAPC in mortality was among females (AAPC=-8.2, 95%CI -9.1 to -7.3). There were greater reductions in the incidence among males in all regions, except for the Central-West. Regarding the mortality rate, Northeast (AAPC=-9.6, 95%CI -10.6 to -8.5), South (AAPC=-8.1, 95%CI -9.8 to -6.3), and Central-West (AAPC=-8.5, 95%CI -10.7 to -6.3) showed their lowest AAPCs among females. There were significant differences in trends between males and females, mostly regarding incidence rates.

**Conclusion:**

Meningitis remains a concerning disease in Brazil, despite the downward trend in the country and its regions. It highlights the perpetuation of health inequalities, which reverberate in the levels of vaccination coverage and in the success of vaccination campaigns, impeding this disease’s proper control.

## RESUMO

**Objetivo:**

Analisar a tendência temporal da incidência, da mortalidade e da letalidade de meningite em crianças e adolescentes de 0-19 anos no Brasil e em suas regiões por sexo de 2002 a 2022. **Métodos:** Trata-se de um estudo de séries temporais dos casos de meningite e óbitos pela doença com dados do Sistema de Informação de Agravos de Notificação. Estimaram-se a variação percentual anual média (VPAM) e seu intervalo de 95% de confiança (IC95%) por regressão *joinpoint*, além de comparar as VPAMs das unidades federativas por teste pareado de coincidência.

**Resultados:**

Houve 274.500 casos e 17.162 óbitos durante o período analisado, com tendência decrescente no Brasil e em suas regiões tanto na incidência quanto na mortalidade. No Brasil, a menor VPAM da incidência foi no sexo masculino (VPAM=-5,7; IC95% -7,1 a -4,2), enquanto o menor VPAM da mortalidade foi no feminino (VPAM=-8,2; IC95% -9,1 a -7,3). Houve maior redução de incidência no sexo masculino, com exceção do centro-Oeste. Quanto à mortalidade, nordeste (VPAM=-9.6; IC95% -10,6 a -8,5), sul (VPAM=-8,1; IC95% -9,8 a -6,3) e centro-oeste (VPAM=-8,5; IC95% -10,7 a -6,3) apresentaram menores VPAMs entre o sexo feminino. Houve diferenças significativas entre as tendências entre os sexos masculino e feminino, sobretudo na incidência.

**Conclusão:**

A meningite ainda é uma doença preocupante no Brasil, apesar das tendências decrescentes no país. Há manutenção de iniquidades na saúde, as quais reverberam na cobertura vacinal e no sucesso das campanhas vacinais, impedindo o controle adequado da doença.

## INTRODUCTION

Meningitis is the inflammation of the nervous system membranes caused by bacteria, viruses, fungi, or noninfectious agents^1^; however, trends in children and teenagers are often overlooked. There are high death rates in this population in Brazil, despite the disease’s inclusion in the Brazilian Compulsory Notification Diseases List^2^. This is enhanced in this population due to blood-brain barrier immaturity, increasing the susceptibility to immediate and late complications, such as cognitive impairment, hearing loss, and death^3^ ^,^ ^4^. High fever, neck stiffness, persistent headache, and lethargy are meningitis’ main signs and symptoms^5^; nonetheless, due to non-specificity, treatment is frequently delayed^6^. Treatment is guided by signs and symptoms, with antibiotic therapy for bacterial agents and supportive therapy for viral etiologies^5^, including *Neisseria meningitidis*, *Streptococcus pneumoniae*, *Haemophilus influenzae* type b (Hib), *Cryptococcus* spp*.*, *Echovirus,* and *Coxsackievirus* ^*5*^.

Globally, there are around 2.5 million cases of meningitis per year, with a higher prevalence in children aged less than 5 years^7^. Additionally, deaths are common, with an estimated number of 236,000 annually, mostly caused by bacterial agents^7^. This etiology is associated with high progression epidemics, especially in the African meningitis belt^7^. In Brazil, there are occasional meningitis outbreaks and epidemics^8^. There were 187,508 cases from 2010 to 2020 with a higher number of cases in 2020^2^ ^,^ ^9^, suggesting inefficient healthcare to restrain cases and deaths. Higher risk of quality-of-life impairment and high costs due to a disorder’s development following meningitis in children and teenagers reveals an economic and public health problem^10^.

Vaccines play a major role in preventing meningitis, as etiologies have different treatment, transmissibility, and severity^11^. In the Brazilian National Immunization Plan (PNI), meningitis vaccinations display irrefutable advances in public health, including the introduction of Hib vaccine in 1999, meningococcal C (MenC) vaccine in 2010, 10-valent pneumococcal vaccine in 2010, 13-valent pneumococcal vaccine in 2019, and meningococcal ACWY vaccine in 2020^11^. However, maintaining high vaccination coverage rates poses important challenges to the PNI^11^, although most common pathogens are currently covered by vaccines, many others are not vaccine-preventable.

Despite its national and global importance, few studies evaluate the trends in meningitis in these populations. Trend analysis helps to identify populations at risk of developing and having the worst outcomes, in addition to providing useful data to plan control of the disease, enhancing the response time and efficiency^3^. Moreover, understanding the temporal patterns encourages further investigations regarding the underlying factors that influence meningitis outbreaks in Brazil.

This study aimed to analyze the trends in incidence, mortality, and lethality rates due to meningitis in children and teenagers aged 0-19 years in Brazil and its regions by sex from 2002 to 2022.

## Methods

### Study design and data sources

This is a time-series study of the cases and deaths due to meningitis in Brazil and its regions, with data from the Notifiable Diseases Information System (SINAN) from 2002 to 2022. We gathered the data in February 2023 and tabulated the data by region and Federative Unit (FU) of residence^12^ ^,^ ^13^. The data were filtered by:

- Region of residence: North, Northeast, Southeast, South and Central-West;
- FU of residence: Acre, Alagoas, Amapá, Amazonas, Bahia, Ceará, Distrito Federal, Espírito Santo, Goiás, Maranhão, Mato Grosso, Mato Grosso do Sul, Minas Gerais, Pará, Paraíba, Paraná, Pernambuco, Piauí, Rio de Janeiro, Rio Grande do Norte, Rio Grande do Sul, Rondônia, Roraima, Santa Catarina, São Paulo, Sergipe e Tocantins;
- First symptom year: 2002-2022;
- Age group (in years): 0-4, 5-9, 10-14, and 15-19;
- Sex: male, female, and both sex;
- Meningitis etiology: bacterial (MB), meningococcemia (MCC), by *Haemophilus influenzae* (MH), meningococcal (MM), meningococcal + meningococcemia (MM+MCC), nonspecific (MNE), by *Streptococcus pneumoniae* (MP), tuberculous (MTBC), viral (MV), and by other etiologies (MOE);
- Case evolution: hospital discharge, death by meningitis, and death by other causes.

Demographic data were gathered from the Information Technology Department of the Unified Health System (DATASUS) and filtered by region of residence, FU of residence, and sex^14^.

### Study participants and variables

We analyzed all cases and deaths due to meningitis among children and teenagers aged 0-19 years in Brazil based on the mandatory notifications in the Unified Health System (SUS)^12^ ^,^ ^13^. We included all notifications from 2002 to 2022 from patients with residence at any Brazilian FUs, in addition to the inclusion of all sexes and etiologies. All ignored and incorrectly tabulated data were excluded from analyses.

We calculated the annual age-adjusted incidence and mortality rates in Brazil and its regions by sex per 100,000 population. The number of new cases or deaths was used as the numerator, whereas the population of a given region was the denominator. The rates were standardized by the 2010 Census’ population in Brazil^14^. We calculated the lethality rates by dividing the number of deaths by the number of new cases for a given year, which was given as a percentage. The number of deaths was given by the evolution of the notified case as death from meningitis in the SINAN system.

### Statistical analysis

We estimated the average annual percent change (AAPC), annual percent change (APC), and its 95% confidence interval (95%CI) by Joinpoint regression^15^. It evaluates whether the data is better fit by a linear model or by multiple segmented linear phases, which are calculated by Monte Carlo permutation. The models produced are compared using the Bayesian information criterion, as the chosen model has the lowest criterion among the other models^15^. The AAPC is the geometric-weighted average of the APCs observed in a given series. Trends were classified as upward, when the AAPC or APC were positive with a positive 95%CI; downward, when the AAPC or APC were negative and a negative 95%CI; and stationary, when the 95%CI encompassed the zero value independently of the AAPC or APC. We pairwise compared the observed trends in each FU by analyzing the existence of disparities in the incidence and mortality trends in male and female populations. We used the coincidence test described by Kim et al.^16^, which evaluated if the regression models produced had identical intercept and slope. Furthermore, trends were classified as noncoincident when p<0.01.

Trend analysis was performed in the Joinpoint v4.9.1.0 software with log-linear data transformation. We used the parametric method to estimate 95%CI with 4,499 repetitions in permutations. We considered the year as the independent variable, whereas the aforementioned rates were the dependent variables. We did not account for autocorrelation, as it could lower statistical power to detect joinpoints^15^; however, the produced models accounted for heteroscedasticity by standard error calculation. Other analyses and plots were performed in R v4.3.0 software with p<0.05 in all analyses, with the exception of the pairwise analysis, in which we considered p<0.01 to reduce type I error possibility^16^.

### Ethical aspects

This study analyzed public access data with secondary and unidentified populations. It was not necessary to obtain ethical approval from an institutional review board; thus, this study is in accordance with the Brazilian National Health Council guidelines.

### Data availability statement

All data supporting the findings of this study are available within the paper.

## RESULTS

There were 274,500 new cases of meningitis, of which 60% occurred in the male population. A total of 17,162 deaths due to meningitis were observed, of which 57% occurred in the male population. Additionally, among both sexes as a whole, the highest age-adjusted incidence rate (34.7/100,000 population) and mortality rate (2.2/100,000 population) observed in Brazil were in 2002, whereas the lowest age-adjusted incidence rate (4.8/100,000 population) and mortality rate (0.3/100,000 population) were observed in 2021. Regarding the Brazilian regions, the highest incidence rate was seen in the South in 2006 (66.8/100,000 population), whereas the highest mortality rate was in the Southeast in 2004 and 2005 with a rate of 2.7/100,000 population (Table 1).

**Table 1.** Annual age-adjusted incidence and mortality rates per 100,000 population due to meningitis in both sexes as a whole by region of residence, Brazil, 2002-2022.

Variable	Year																				
	2002	2003	2004	2005	2006	2007	2008	2009	2010	2011	2012	2013	2014	2015	2016	2017	2018	2019	2020	2021	2022
Incidence rate																					
Brazil	34.7	24.4	24.1	24.9	27.5	31.7	23.3	20.4	18.4	18.6	20.4	17.4	15.8	13.9	14.0	15.3	15.8	15.0	5.7	4.8	10.1
North	14.2	12.9	11.1	9.8	10.1	8.2	8.7	7.8	5.7	5.9	5.4	5.9	5.7	6.0	5.2	5.6	5.4	4.6	2.9	2.3	3.3
Northeast	18.5	15.9	14.2	14.4	12.7	25.8	20.5	16.6	11.9	13.0	14.8	11.4	8.4	7.2	5.6	6.7	6.2	6.6	2.7	2.3	5.5
Southeast	51.0	32.7	33.7	31.7	32.2	38.6	28.8	26.5	26.9	26.1	31.1	25.6	24.9	19.7	22.5	22.2	25.8	22.6	7.7	6.4	14.9
South	49.9	31.8	34.0	45.8	66.8	52.8	30.6	26.4	23.9	25.2	20.6	22.1	20.3	23.1	19.8	28.3	24.1	26.7	10.7	9.4	14.9
Central-West	17.4	18.7	16.3	18.1	20.5	16.5	14.4	13.1	11.2	11.0	12.1	9.7	8.3	8.0	8.1	7.9	6.0	6.8	3.7	3.4	4.7
Mortality rate																					
Brazil	2.2	2.2	2.1	2.0	1.9	1.4	1.3	1.5	1.3	1.2	1.0	0.9	0.8	0.7	0.7	0.7	0.7	0.7	0.4	0.3	0.5
North	1.6	2.2	1.3	1.3	1.4	0.9	0.9	1.1	0.9	0.8	0.5	0.7	0.6	0.7	0.6	0.5	0.4	0.6	0.5	0.3	0.3
Northeast	1.9	1.8	1.7	1.6	1.4	1.1	1.0	1.2	0.8	0.9	0.7	0.6	0.5	0.5	0.5	0.4	0.5	0.6	0.3	0.3	0.4
Southeast	2.6	2.6	2.7	2.7	2.4	1.7	1.7	2.0	2.1	1.6	1.5	1.3	1.1	0.9	0.9	0.9	0.9	0.8	0.4	0.4	0.7
South	2.2	2.2	2.3	1.8	2.1	1.3	1.3	1.2	0.9	0.9	0.8	1.0	0.7	0.9	0.8	0.8	0.8	0.8	0.3	0.4	0.6
Central-West	1.6	2.1	1.6	1.9	1.5	1.4	1.5	1.3	1.3	0.9	0.9	0.9	0.9	0.6	0.7	0.7	0.5	0.7	0.3	0.3	0.5

We only found downward trends, both when analyzing the country and its regions, in addition to downward trends in all sexes analyzed. In Brazil, the male population had the greatest reduction (AAPC=-5.7, 95%CI -7.1 to -4.2) among all sexes when we analyzed the age-adjusted incidence rates (Table 2). When the age-adjusted mortality rates were analyzed, the female population had the greatest reduction (AAPC=-8.2, 95%CI -9.1 to -7.3) among all sexes (Table 3).

**Table 2.** Trends in the age-adjusted incidence rate per 100,000 population due to meningitis by sex and region of residence, Brazil, 2002-2022.

Variable	Period	APC^†^ (95%CI^‡^)	Trend (period)	AAPC^§^ (95%CI^‡^)	Trend (2002-2022)
Both sex					
Brazil	2002-2022	-5.6 (-7.0 to -4.1)*	D	-5.6 (-7.0 to -4.1)*	D
North	2002-2011	-9.1 (-11.3 to -6.9)*	D	-8.1 (-11.1 to -5.0)*	D
	2011-2018	-1.6 (-7.3-4.5)	S		
	2018-2022	-16.4 (-27.1 to -4.0)*	D		
Northeast	2002-2007	6.6 (-2.7-16.7)	S	-6.7 (-9.2 to -4.1)*	D
	2007-2022	-10.7 (-12.9 to -8.4)*	D		
Southeast	2002-2022	-4.7 (-6.3 to -3.1)*	D	-4.7 (-6.3 to -3.1)*	D
South	2002-2022	-5.9 (-8.2 to -3.5)*	D	-5.9 (-8.2 to -3.5)*	D
Central-West	2002-2006	1.2 (-6.3-9.3)	S	-6.8 (-8.4 to -5.1)*	D
	2006-2022	-8.7 (-10.0 to -7.4)*	D		
Male					
Brazil	2002-2022	-5.7 (-7.1 to -4.2)*	D	-5.7 (-7.1 to -4.2)*	D
North	2002-2011	-9.2 (-11.6 to -6.8)*	D	-8.4 (-11.7 to -5.1)*	D
	2011-2018	-1.4 (-7.6-5.3)	S		
	2018-2022	-18.0 (-29.4 to -4.6)*	D		
Northeast	2002-2008	4.5 (-4.4-14.2)	S	-7.0 (-10.4 to -3.5)*	D
	2008-2022	-11.5 (-15.2 to -7.7)*	D		
Southeast	2002-2022	-4.9 (-6.5 to -3.3)*	D	-4.9 (-6.5 to -3.3)*	D
South	2002-2022	-6.0 (-8.3 to -3.6)*	D	-6.0 (-8.3 to -3.6)*	D
Central-West	2002-2006	1.9 (-6.5-11.1)	S	-6.6 (-8.4 to -4.8)*	D
	2006-2022	-8.6 (-10.0 to -7.2)*	D		
Female					
Brazil	2002-2022	-5.4 (-6.8-4.0)*	D	-5.4 (-6.8 to -4.0)*	D
North	2002-2022	-6.6 (-7.6 to -5.6)*	D	-6.6 (-7.6 to -5.6)*	D
Northeast	2002-2007	5.6 (-3.5-15.6)	S	-6.8 (-9.3 to -4.2)*	D
	2007-2022	-10.6 (-12.8 to -8.4)*	D		
Southeast	2002-2022	-4.5 (-6.0 to -2.9)*	D	-4.5 (-6.0 to -2.9)*	D
South	2002-2022	-5.7 (-8.1 to -3.3)*	D	-5.7 (-8.1 to -3.3)*	D
Central-West	2002-2006	0.3 (-7.7-9.0)	S	-7.0 (-8.8 to -5.2)*	D
	2006-2022	-8.7 (-10.1 to -7.3)*	D		

^†^Annual percent change; ^‡^95% confidence interval; ^§^Average annual percent change; *p<0.05; D: downward; S: stationary.

**Table 3.** Trends in the age-adjusted mortality rate per 100,000 population due to meningitis by sex and region of residence, Brazil, 2002-2022.

Variable	Period	APC^†^ (95%CI^‡^)	Trend (period)	AAPC^§^ (95%CI^‡^)	Trend (2002-2022)
Both sex					
Brazil	2002-2022	-8.1 (-8.9 to -7.2)*	D	-8.1 (-8.9 to -7.2)*	D
North	2002-2022	-7.8 (-9.2 to -6.4)*	D	-7.8 (-9.2 to -6.4)*	D
Northeast	2002-2022	-9.0 (-10.0 to -8.1)*	S	-9.0 (-10.0 to -8.1)*	D
Southeast	2002-2022	-7.8 (-9.0 to -6.6)*	D	-7.8 (-9.0 to -6.6)*	D
South	2002-2022	-7.7 (-9.1 to -6.3)*	D	-7.7 (-9.1 to -6.3)*	D
Central-West	2002-2022	-7.4 (-8.7 to -6.1)*	S	-7.4 (-8.7 to -6.1)*	D
Male					
Brazil	2002-2022	-7.9 (-8.8 to -7.0)*	D	-7.9 (-8.8 to -7.0)*	D
North	2002-2022	-8.6 (-10.3 to -7.0)*	D	-8.6 (-10.3 to -7.0)*	D
Northeast	2002-2022	-8.5 (-9.7 to -7.3)*	S	-8.5 (-9.7 to -7.3)*	D
Southeast	2002-2010	-4.6 (-7.9 to -1.2)*	D	-8.3 (-10.3 to -6.2)*	D
	2010-2022	-10.6 (-13.5 to -7.7)*			
South	2002-2022	-7.4 (-8.7 to -6.0)*	D	-7.4 (-8.7 to -6.0)*	D
Central-West	2002-2022	-6.5 (-8.0 to -5.0)*	S	-6.5 (-8.0 to -5.0)*	D
Female					
Brazil	2002-2022	-8.2 (-9.1 to -7.3)*	D	-8.2 (-9.1 to -7.3)*	D
North	2002-2022	-6.6 (-8.5 to -4.8)*	D	-6.6 (-8.5 to -4.8)*	D
Northeast	2002-2022	-9.6 (-10.6 to -8.5)*	S	-9.6 (-10.6 to -8.5)*	D
Southeast	2002-2022	-7.8 (-9.0 to -6.5)*	D	-7.8 (-9.0 to -6.5)*	D
South	2002-2022	-8.1 (-9.8 to -6.3)*	D	-8.1 (-9.8 to -6.3)*	D
Central-West	2002-2022	-8.5 (-10.7 to -6.3)*	S	-8.5 (-10.7 to -6.3)*	D

^†^Annual percent change; ^‡^95% confidence interval; ^§^Average annual percent change; *p<0.05; D: downward; S: stationary.

All regions showed greater reductions in the age-adjusted incidence rate in the male population with the exception of the Central-West region, which had an AAPC of -6.6 (95%CI -8.4 to -4.8) among males and -7.0 (95%CI -8.8 to -5.2) among females. Regarding mortality rates, only the North (AAPC=-8.6, 95%CI -10.3 to -7.0) and Southeast (AAPC=-8.3, 95%CI -10.3 to -6.2) had greater reductions among the female population. Additional information regarding trends in regions and their respective AAPC or APC is described in Tables 2 and 3.

**Figure 1:**
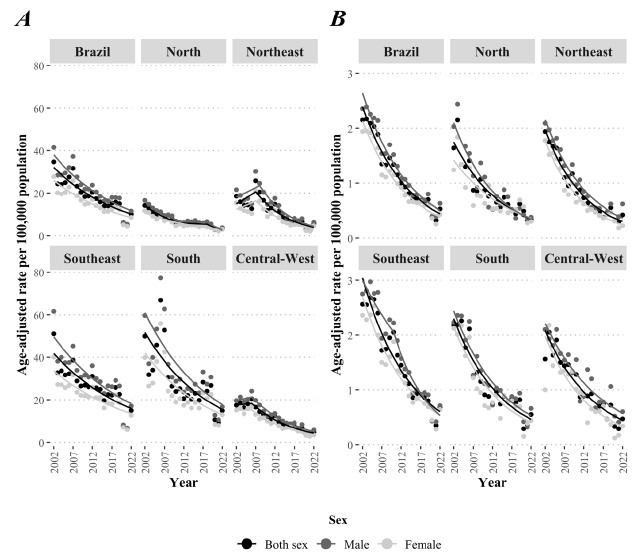
(A) Temporal series of the age-adjusted incidence rates and (B) the age-adjusted mortality rates per 100,000 population due to meningitis according to the region of .residence and sex, Brazil, 2002-2022.

A few regions had joinpoints in their incidence rate trends within the period of analysis. When analyzing both sexes as a unit, the North region had a joinpoint in 2011 and 2018, with a downward trend from 2002 to 2011 (APC=-9.1, 95%CI -11.3 to -6.9), stationary from 2011 to 2018 (APC=-1.6, 95%CI -7.3-4.3), and downward again from 2018 to 2022 (APC=-16.4, 95%CI -27.1 to -4.0). In the Northeast, there were two distinct trends: a stationary one from 2002 to 2007 (APC=6.6, 95%CI -2.7-16.7) and a downward one from 2007 to 2022 (APC=-10.7, 95%CI -12.9 to -8.4). In the Central-West, a stationary trend was observed from 2002 to 2006 (APC=1.2, 95%CI -6.3-9.3) and a downward trend from 2006 to 2022 (APC=-8.7, 95%CI -10.0 to -7.4) (Figure 1A).

Regarding the trends in the age-adjusted mortality rates, only the Southeast region had a joinpoint among males in 2010, dividing it into two trends. Both trends were downward; however, we observed an intensification of the initial APC of -4.6 (95%CI -7.9 to -1.2) from 2002 to 2010 compared to the final one of -10.6 (95%CI -13.5 to -7.7) from 2010 to 2022 (Figure 1B). Additional information regarding the joinpoints observed among the series analyzed is shown in Figure 1.

**Figure 2.**
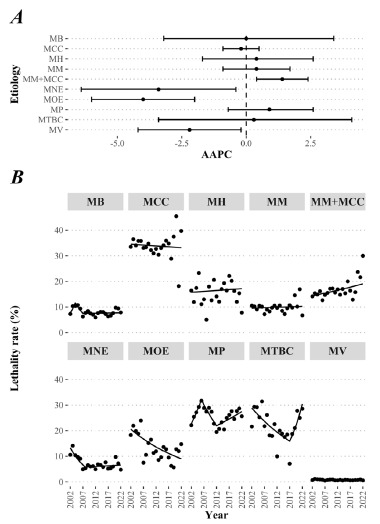
(A) Temporal series of the lethality rates due to meningitis and (B) their respective trends according to the etiology, Brazil, 2002-2022.

AAPC: average annual percent change; MB: bacterial; MCC: meningococcemia; MH: *Haemophilus influenzae*; MM: meningococcal; MM+MMC: meningococcal + meningococcemia; MNE: nonspecific; MOE: other etiologies; MP: *Streptococcus pneumoniae*; MTBC: tuberculous; MV: viral.

We found higher lethality rates in the meningococcemia etiology, with the highest one being in 2020 (45.0%). Only the MM+MCC etiology had an upward trend (AAPC=1.4, 95%CI 0.4-2.4), while the MNE (AAPC=-3.4, 95%CI -6.4 to -0.4), MOE (AAPC=-4.0, 95%CI -6.0 to -2.0), and MV (AAPC=-2.2, 95%CI -4.2 to -0.2) had downward trends (Figure 2A).

There were joinpoints in several etiology series analyzed. For instance, MB displayed three distinct trends: 2002-2004 (APC=23.1, 95%CI 2.5-47.9), 2004-2007 (APC=-13.5, 95%CI -28.4-4.4), and 2007-2022 (APC=0.2, 95%CI -1.4-1.8). In the MNE etiology, the trend was initially downward from 2002 to 2008 (APC=-12.9, 95%CI -19.9 to -5.3) and then stationary from 2008 to 2022 (APC=0.9, 95%CI -2.3-4.3). Moreover, in the MP, there were three distinct trends: an upward trend from 2002 to 2006 (APC=8.9, 95%CI 3.9-14.3), a downward trend from 2006 to 2012 (APC=-6.1, 95%CI -9.6 to -2.4), and an upward trend from 2012 to 2022 (APC=2.3, 95%CI 0.3-4.3). Lastly, in the MTBC etiology, a single joinpoint was found in 2017 that divided the trend into an initial downward trend (APC=-3.9, 95%CI -6.1 to -1.6) and a final stationary trend (APC=13.8, 95%CI -1.8-31.9) (Figure 2B). All the other analyses about trends regarding the etiologies of meningitis are plotted in Figure 2.

**Figure 3.**
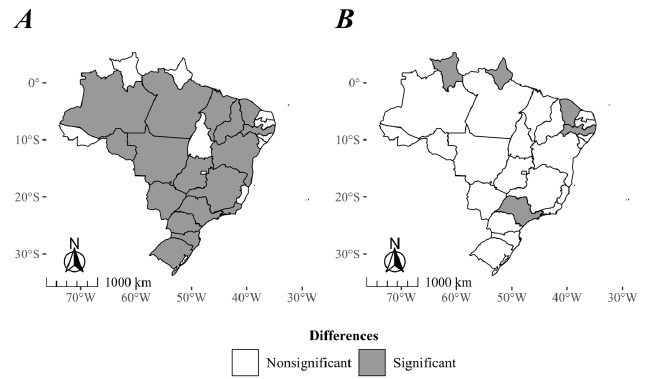
Differences in the trends of (A) the age-adjusted incidence and (B) mortality rates due to meningitis between males and females according to the pairwise coincide test, Brazil, 2002-2022.

Finally, the differences found when we compared the incidence rate trends between male and female populations are described in Figure 3. Acre, Alagoas, Amapá, Distrito Federal, Espírito Santo, Paraíba, Rio Grande do Norte, Roraima, Sergipe, and Tocantins had coincident trends among male and female populations. All other FUs had noncoincident trends and showed greater AAPCs among the male population, in which the highest AAPC differences were in Rondônia (AAPC=4.5, 95%CI 1.1-7.9), followed by Mato Grosso do Sul (AAPC-4.2, 95%CI 1.6-6.8), Piauí (AAPC-3.9, 95%CI -2.9-10.7), and Mato Grosso (AAPC=3.8, 95%CI 0.7-6.9) (Figure 3A).

In regard to the differences in the mortality rate between males and females, only Roraima (AAPC=10.4, 95%CI 6.2-14.6), Ceará (AAPC=7.7, 95%CI 4.2-11.2), Pernambuco (AAPC=4.3, 95%CI 1.4-7.1), and São Paulo (AAPC=3.3, 95%CI 1.3-5.3) showed differences. These FUs showed an upward trend in the difference between males’ and females’ mortality rates, whereas only Amapá (AAPC--7.5, 95%CI -0.9 to -14.2) showed higher AAPC in females, with an upward trend in the difference (Figure 3B).

## DISCUSSION

We analyzed the trends in incidence, mortality, and lethality rates due to meningitis in Brazil and its regions by sex. We found downward trends in incidence and mortality age-adjusted rates in all series, with greater reductions in incidence rates in males and greater reductions in mortality rates in females. Nevertheless, trends concerning the analyzed etiologies varied, even though most of them were stationary. When male and female trends in FUs were compared, there were significant differences especially in incidence rates.

Trends in incidence and mortality due to meningitis were downward in Brazil. This is in accordance with other studies^17^ ^,^ ^18^ ^,^ ^19^ ^,^ ^20^ ^,^ ^21^, as a global study conducted from 1990 to 2019 showed a 38% and a 62% overall decrease in incidence and mortality rates among children aged less than 5 years, respectively^17^. Our study agrees with the national literature^19^ ^,^ ^21^, in which reductions in incidence and mortality rates due to meningitis are seen without considering the influence of meningitis etiologies. A successful vaccination, especially the Hib, pneumococcal, and meningococcal^17^ ^,^ ^18^ ^,^ ^22^, explains our results, as it provides greater herd immunity and greater reduction in nasopharynx pathogen carriage^18^ ^,^ ^23^. Polysaccharide vaccines exhibit a diminished response in serogroup C meningococcal disease in comparison to the conjugated vaccine^18^ ^,^ ^23^. Moreover, carriage rates, vaccine types, and disease prevalence may influence the level of vaccination required to achieve herd immunity^18^.

Yet, in spite of the downward trends, Brazilian regions differed regarding incidence and mortality rates. A national study from 2005 to 2018 revealed a decline in the MenC vaccination rate from 99% in 2013 to 83% in 2018, with lower uptakes in the North region^19^, indicating varying vaccination coverages. Furthermore, adverse economic conditions, low educational level, and lack of awareness of disease and vaccines, in addition to religious and cultural beliefs, impede proper vaccination^24^. It is explained by the per capita expenditure on education in association with vaccination coverage, in which economic, health, education, and demographic structure are key to maintaining an optimal coverage^22^. Unequal access to vaccines and healthcare facilities inhibits appropriate vaccination, as there are lower vaccination coverages in underdeveloped locations in comparison to the richest regions in Brazil^25^ ^,^ ^26^.

All regions showed reductions in incidence and mortality rates with variable AAPC values. In addition to the aforementioned factors, this could be attributed to organizational barriers, which are common in most underdeveloped regions, encompassing the availability of vaccines, the upkeep of immunization records, and the coordination with community resources^24^. This provokes a higher tendency toward vaccine hesitancy, which is correlated with a lack of confidence, complacency, and recommendation, thereby reducing the search for vaccines^24^. However, cultural and dietary factors should be considered, as raw food consumption may be associated with the onset of eosinophilic meningitis outbreaks, as some animals might be infected with a nematode that lodges in the central nervous system^27^.

There are persistent rates both in incidence and mortality rates, irrespective of the reductions in AAPC in all regions and in Brazil as a whole. A global study reported a mortality rate of 16.9/100,000 in 2019^17^, whereas a Brazilian study showed a mortality rate of 1.4/100,000 in infants aged less than 1 year after 2010^19^. The persistence of these elevated rates can be attributed to the multipathogenic nature of meningitis, wherein only a limited number of pathogens are currently vaccine-preventable, resulting in a slower decline in mortality among infants under the age of 5 years^17^. Nevertheless, the introduction of Hib vaccination in 1999 reduced this disease cases and deaths^28^; therefore, it appears that other serogroups of *Haemophilus influenzae* and *Streptococcus pneumoniae* are accounting for the remaining ecological niche^29^.

The meningococcal etiology had higher lethality rates among all other etiologies, regardless of stationary trends observed in Brazil. This etiology has the potential for sudden onset and rapid progression, as patients may not necessarily exhibit signs of meningeal irritation, thereby complicating the diagnosis and leading to unwanted adverse outcomes^30^. Despite the high lethality rates, there was a significant and consistent decline in meningococcal disease, subsequent to the inclusion of vaccines in the PNI, resulting in a reduction in serogroup C incidence, particularly among children under the age of 5 years after 2010 compared with periods before vaccine introduction^19^. After this, serogroup B emerged as the most prevalent within this population^19^. Nevertheless, the prevalence of serogroup W in children under the age of 1 year appears to be increasing, particularly in the South region^19^. Yet, MP and meningitis caused by Hib had overall stationary trends in lethality rates. However, MP had a downward trend from 2006 to 2012, encompassing the year that the pneumococcal vaccine was introduced^11^, while the upward trend from 2012 to 2022 seen may be linked to decreasing vaccination coverage rates for this vaccine or other serogroups^29^.

Meningococcal etiology had a stationary trend in our study from 2002 to 2022. Despite this, the COVID-19 pandemic had a negative impact on the number of MenC vaccine doses applied^31^, increasing the number of susceptible individuals and aggravating pre-existing regional inequalities in vaccination coverage^31^. Moreover, this led to an underreporting of meningitis cases and deaths during the initial phase of the pandemic, as a result of fewer diagnoses or the inability to accurately account for meningitis cases or deaths in the surveillance system^32^. The historical heterogeneity of Brazilian healthcare conditions among its regions and the increase in social inequalities and vaccination coverage suggest the North region to be one of the most affected by underreporting^31^. Yet, it was not possible to assess the impact of the ACWY vaccine due to an overlap of its implementation and the onset of the pandemic in 2020.

Furthermore, trends in incidence and mortality rates due to meningitis among the FUs differed, mostly in the incidence rate. This could be attributed to the sex bias in infectious diseases^33^, as a study conducted in Brazil revealed a modest but significant male bias in the incidence and mortality rates of meningitis in infants. There are two hypotheses: the physiological, which encompasses organic differences between males and females; and the behavioral, which encompasses exposure differences between males and females^33^. However, the first hypothesis holds greater plausibility^33^ ^,^ ^34^, as estrogen receptors signaling regulates immune cell functions^34^. Additionally, this signaling expressed by microglia promotes an anti-inflammatory response to central nervous system conditions, resulting in neuroprotection in those types of injuries^34^. These receptors enhance the immune response and outcomes within the female population^34^.

The differences between trends in male and female populations show greater male involvement in incidence and mortality rates. In addition to the male bias^33^, females produce more antibodies following vaccinations than males, as reduced concentrations of estradiol are related to reduced vaccine-induced immunity^34^. The signaling of androgen receptors, which is more common in males, causes differences in the humoral immune response, reducing antibody response and efficiency of B cells in this population^34^.

This study is not free of limitations, and the primary ones are related to secondary database utilization and its design, as underreporting could be a factor^19^ ^,^ ^21^ ^,^ ^32^, as well as the inability to conduct individual-level analysis, respectively. It is possible that the database underestimates the actual number of cases, as it could be attached to correct surveillance in each municipality^21^, in addition to the influence that the COVID-19 pandemic could have had on these notifications^32^. Moreover, analyzing countries with extensive territory, such as Brazil, poses limitations, including the heterogeneity between its regions, FUs, and populations; however, we performed our analysis according to the FUs, regions, and sex to minimize this limitation. Finally, meningitis has several etiologies with different pathogens, disease courses, transmissibility, and severity. Nevertheless, we analyzed the data per etiology to minimize this limitation.

Despite these limitations, our study provides current estimates of the incidence, mortality, and lethality rates due to meningitis in Brazil and its regions by sex in children and teenagers aged 0-19 years. These estimates are essential to understanding the current disease scenario in Brazil, as we presented age-adjusted rates for all measured outcomes, in addition to lethality rates trend analysis. To our knowledge, this is the first study to conduct a Joinpoint analysis of the lethality rates due to meningitis, providing valuable information for further studies. Additionally, our study helps to identify the most susceptible regions and populations to this disease, in addition to providing further data that support the physiological hypothesis regarding male bias in infectious diseases^33^ ^,^ ^34^. Our findings could be useful when developing new measures to control meningitis and to improve surveillance in regions that did not show adequate disease control.

In conclusion, meningitis remains a major concern in Brazilian territory. Regardless of the downward trends, there are persistent high incidence, mortality, and lethality rates in most regions. Moreover, vaccination campaigns among Brazilian regions, as their populations show different levels of vaccine coverage, highlight social inequalities and varied levels of commitment in controlling this disease. It is imperative that meningitis control encompass not only the reduction of social inequalities, but also the enhancement of healthcare, vaccines, and information accessibility in underprivileged areas and populations.
